# Revisions of Theophanes Chrysobalantes *De Curatione*

**DOI:** 10.12688/wellcomeopenres.9991.2

**Published:** 2017-04-10

**Authors:** Barbara Zipser

**Affiliations:** 1 Department of History, Royal Holloway University of London, Egham/Surrey, UK

**Keywords:** Theophanes Chrysobalantes' De curatione, humanities, translation

## Abstract

**Background:** Theophanes Chrysobalantes' *De curatione* is a little known but highly relevant therapeutic manual dating to the tenth century AD. The text has come down to us in  an unusually large number of manuscripts, most of which transmit a mainstream version of the text. **Methods:** In the present article, three versions deriving from the mainstream text are being examined. For this, these versions are being compared to the mainstream text, in order to understand the aim behind the alterations and additions they were subjected to. The overarching goal is to understand, why these changes were made, and how skilled the editors were. It is a rather unusual approach, as divergent versions are usually not examined in research literature, since they are secondary to the original text. **Results and conclusions:** The results clearly show that the text was redacted several times, but not by highly sophisticated editors. The general aims of the redactions were to make the text easier to understand.

## Introduction

It ^1^ is not unusual for Byzantine medical texts to be revised by editors – but mostly, these were revisions of texts that were corrupted in the course of transmission, for instance if a manuscript was damaged ^2^. Here, the main aim of the editor was obviously to restore the text to its original form. This article focusses on a completely different type of revision, namely a rephrased, augmented or otherwise heavily altered text. These are not that common as far as standard medical manuals are concerned, and they are not usually the focus of scholarly analysis.

The samples that are going to be discussed below come from manuscripts transmitting Theophanes Chrysobalantes *De Curatione*, a little known but highly relevant therapeutic manual that was in its mainstream form composed in the tenth century AD ^3^. This work contains a substantial collection of therapeutic instructions arranged in a head to foot order, along with some miscellaneous material at the end. During my research on the transmission of the work I found that four manuscripts transmit a significantly altered text compared to the mainstream text. These are in alphabetical order ^4^:

- Escorial, Real Biblioteca, T III 1, 16 ^th^ century, f. 1r-141v ^5^.
- Florence, Biblioteca Laurenziana Plut. 75, 6, 14 ^th^ century, f. 1r-54v ^6^.
- London, Wellcome Library MSL 135, 16 ^th^ century, f. 1r-86r ^7^.
- Palermo, Biblioteca Centrale della Regione Siciliana XIII C 3, 16 ^th^ century, f. 121r-149r ^8^.

The manuscripts from Florence and the Wellcome Library present a near identical text, and are therefore closely related and probably siblings ^9^. So in total, we are dealing with three versions of *De Curatione*, which differ significantly from the mainstream tradition. In the following, I am going to describe these three versions very briefly. The main research question to be addressed is, what purpose these alterations served.

## Sources

Starting, or rather collating, from the beginning of *De Curatione*, the first chapter showing significant discrepancies from the mainstream text is chapter 4 (Bernard). As it is common for Byzantine therapeutic texts, the chapter starts with a heading and a brief description of a disease, which is then followed by several alternative recipes for medical treatment. Generally, in this genre, the descriptive part of the chapter tends to have a stable transmission, whereas the transmission of the recipes is notoriously unstable ^10^. In this case, however, the descriptive parts also show significant differences, and since this is unusual, these will form the basis for this article.

Below, I present a comparison of the mainstream text as edited in the Bernard edition, and two versions, the Florence/Wellcome group and the Escorial version. The Palermo manuscript will be discussed at a later point in the present article, as it does not show any significant variants against the mainstream version in this part of *De Curatione* as far as the text itself is concerned. Rather, it is the selection and organisation of excerpts that is of interest.

### Edition of the mainstream text (Bernard)

4. Περὶ πιτυριάσεως. ἡ πιτυρίασις λεπτῶν καὶ πιτυροειδῶν σωμάτων ἐκ τῆς ἐπιφανείας τῆς κεφαλῆς καὶ τοῦ ἄλλου σώματός ἐστιν ἀπότηξις χωρὶς ἑλκώσεως. γίνεται δὲ καὶ κακοχυμίας ἀνενεχθείσης ^11^ παρὰ τὴν κεφαλὴν ἢ φλέγματος ἁλμυροῦ ἢ χολώδους καὶ μελαγχολικοῦ αἵματος. καὶ δεῖ κενοῦν πρῶτον τὸ ὅλον σώμα διὰ φλεβοτόμου καὶ καθάρσεως τοῦ πλεονεκτοῦντος χυμοῦ. ἔπειτα κιμωλίαν ἀποβρέξας ὕδατι, μίξον σεύτλου χυλὸν καὶ κατάχριε.

Translation: On pityriasis. Pityriasis is a melting of smooth and bran like bodies from the surface of the head or of the rest of the body, without rupturing. It happens when bad humours are brought up around the head either consisting of salty phlegm or bilious and melancholic blood. And one first needs to empty the whole body through blood letting and evacuation of the superfluous humour. Then rinse earth from Cimolus with water, mix with beet root juice and apply.

8. Περὶ ἀλωπεκίας καὶ ὀφιάσεως. Ἀλωπεκία ἐστιν ὅταν αἱ τρίχες ἀποπέσωσι τῆς κεφαλῆς διὰ χυμὸν θερμὸν καὶ διαβρωτικὸν κόπτοντα τὰς ῥίζας αὐτῶν. Διαφέρει δὲ ἡ ὀφίασις τῷ σχήματι. Ἡ γὰρ ὀφίασις λειοτέρα ἐστὶν ὥσπερ οἱ ὄφεις. Ἡ δὲ ἀλωπεκία τραχυτέρα πολλῷ. Στοχάζου δὲ ἀπὸ τῆς χροιᾶς τοῦ δέρματος τὸν πλεονάζοντα χυμὸν καὶ τούτου τὴν κάθαρσιν πρῶτον ποιοῦ. Εἰ μὲν ἐπὶ τὸ μελάντερον ἢ λευκότερόν ἐστιν ἡ χροιὰ διὰ τῆς ἱερᾶς ἀντιδότου καθαίρομεν. Εἰ δὲ ἐπὶ τὸ ὠχρότερον (κίτρινον Ro1 ^12^) τὰ διὰ τῆς ἀλόης καὶ τῆς πικρᾶς...

On alopecia and ophiasis. Alopecia is when the hair of the head falls out because of a hot and corrosive humour cutting off the roots. Ophiasis has a different form. For ophiasis is smoother like snakes. Alopecia is far more rough. Assess the superfluous humour from the colour of the skin, and first remove it. If the colour is towards more black or white, we purge with the holy (antidote). If it is paler ^13^ (yellow Ro1), the aloe- and bitter [remedies]…

### Transcription of the Florence/Wellcome group

The text given below has been transcribed from Plut. 75, 6 (MSL 135 presents an almost identical text with just a few additional minor mistakes, which are irrelevant to the argument, and has therefore not been included) ^14^:

4. Περὶ πιτυριάσεως. Ἡ πιτυρίδα γίνεται μὲν ἐπὶ πλέον εἰς τὴν κεφαλήν. Γίνεται δὲ καὶ εἰς τὸ ἄλλο δέρμα τῆς σαρκὸς τῆς λευκῆς καὶ πιτυροειδοῦς χωρὶς ἑλκώσεως. Γίνεται δὲ ὅταν ἀνέλθη ὕλη τις εἰς τὴν κεφαλὴν ἢ ἀπὸ φλέγματος ἁλμυροῦ ἢ χολώδους καὶ μελαγχολικοῦ αἵματος. Καὶ πρέπει πρῶτον ἵνα κενώσης ἀπὸ τοῦ ὅλου σώματος τὸν πλεονάζοντα χυμὸν καὶ διὰ φλεβοτομίας καὶ ἑτέρας καθάρσεως. Ἔπειτα κιμωλίαν βρέξας ὕδατι μίξον καὶ σεύτλου ζωμὸν καὶ ἄλειφε.

Translation: On pityriasis. Pityriasis happens mainly on the head. It also happens on the other white and bran like skin of the flesh without rupturing. It happens when some matter goes up into the head or from salty phlegm or bilious and melancholic blood. And first you should empty the superfluous humour from the whole body both through blood letting and other removal. Then rinse earth from Cimolus with water, mix also beet root juice and apply.

8. Περὶ ἀλωπεκίας καὶ ὀφιάσεως. Ἀλωπεκία ἐστίν, ὅταν αἱ τρίχες ἀποπέσωσι τῆς κεφαλῆς ἀπὸ αἰτίας θερμῶν χυμῶν καὶ διαβρωτικῶν τρωγόντων καὶ κοπτόντων τὰς ῥίζας τῶν τριχῶν. Διαφέρει δὲ καὶ ἔχει ἐναλλαγὴν ἡ ὀφίασις κατὰ τὸ σχῆμα. Ἡ γὰρ ὀφίασις λειοτέρα ἐστι καὶ ὁμαλωτέρα ὥσπερ οἱ ὄφεις. Ἡ δὲ ἀλωπεκία ἐστι τραχεῖα. Στοχάζου δὲ ἀπὸ τοῦ χρώματος τοῦ δέρματος τὸν πλεονάζοντα χυμὸν καὶ ποίει πρῶτον τούτου τὴν ἐκβολὴν καὶ τὴν κάθαρσιν. Καὶ εἰ μὲν ἔστι τὸ χρῶμα πρὸς τὸ μελανώτερον ἢ πρὸς λευκώτερον ποιοῦ μὲν τὴν κάθαρσιν διὰ τῆς ἱερᾶς ἀντιδότου. Εἰ δὲ πρὸς τὸ ὠχρότερον καὶ κιτρινώτερον καθαίρομεν διὰ τῆς ἀλόης καὶ τῆς πικρᾶς…

Translation: On alopecia and ophiasis. Alopecia is when the hair of the head falls off because of an aetiology of hot and corrosive eating away humours that cut off the roots of the hair. Ophiasis differs and diverges according to its form. For ophiasis is smoother and more even like snakes. Alopecia is rough. Assess the superfluous humour from the colour of the skin, and first expel and remove it. And if the colour is more towards black or towards white, remove it through the holy antidote. And if it is more towards paler and yellower, we remove it through the aloe- and the bitter [remedy]…

### Transcription of T III 1, the Escorial manuscript:

4. Περὶ πιτυριάσεως. Ἡ πιτυρίασις λεπτῶν καὶ πιτυροειδῶν λεπιδίων ἐκ τῆς ἐπιφανείας τῆς κεφαλῆς καὶ τοῦ λοιποῦ σώματος ἀπότεξις ἐστὶν ἄνευ ἑλκώσεως. Γίνεται δὲ ἐκ κακοχυμίας ἀνενεχθείσης περὶ τὴν κεφαλὴν ἢ φλέγματος ἁλμυροῦ ἢ χολώδους καὶ μελαγχολικοῦ αἵματος. Καὶ πρῶτον δεῖ τὸν ἰατρὸν κενοῦν τὸ σῶμα ἐκ τοῦ πλεονεκτούντος χυμοῦ διὰ φλεβοτομίας ἢ καθάρσεως προσηκούσης τῷ χυμῷ. Εἶτα χρῆσθαι ἀλείμμασιν. Ἔσεται δὲ ἄλειμμα ἡ κιμωλία προσβραχεῖσα ὕδατι καὶ τεύτλῳ χυμῷ μιχθεῖσα ἀλειφομένη.

Translation: On pityriasis. Pityriasis is a melting of smooth and bran like scales from the periphery of the head and the rest of the body without rupturing. It happens from bad humours that are brought up around the head or from salty phlegm or bilious and melancholic blood. And first the doctor has to empty the superfluous humour from the body through blood letting or a form of purging that is appropriate for the humour. Then to use salves. The salve will be earth from Cimolus rinsed with water and mixed with beet root juice applied.

8. Περὶ ἀλωπεκίας καὶ ὀφιάσεως. Ἀλωπεκία ἐστι μάδησις τριχῶν κεφαλῆς, ἀποπίπτουσι δὲ αἱ τρίχες διὰ χυμὸν θερμὸν καὶ διαβρωτικὸν τὰς ῥίζας αὐτῶν ἀποσπῶντα. Διαφέρει δὲ ἡ ὀφίασις τῷ σχήματι. Ἡ γὰρ ὀφίασις λειοτέρα ἐστὶν ὥσπερ οἱ ὄφεις. Ἡ δὲ ἀλωπεκία τραχυτέρα πολλῷ. Στοχάζου δὲ ἀπὸ τῆς τοῦ δέρματος χροιᾶς τὸν πλεονάζοντα χυμὸν καὶ τούτου τὴν κάθαρσιν πρῶτον ποιοῦ. Εἰ μὲν γὰρ ἐπὶ τὸ μελάντερον ἢ λευκότερον ἐστὶν ἡ χροιὰ διὰ τῆς ἱερᾶς καθαίρομεν. Εἰ δὲ ἐπὶ τὸ ὠχρότερον διὰ τῆς ἀλόης καὶ τῆς πικρᾶς…

Translation: On alopecia and ophiasis. Alopecia is baldness of the hair of the head. The hair falls off because of a hot and corrosive humour that detaches their roots. Ophiasis has a different form, for ophiasis is smoother like snakes. Alopecia is much rougher. Assess the superfluous humour from the colour of the skin, and first remove it. For if the colour is more towards blacker or whiter, we clean it through the Holy [remedy]. If it is towards the paler, through the aloe- and the bitter [remedy]…

## Discussion

In the samples presented above, the transmission of the mainstream text is fairly consistent, and also sufficiently consistent with the Bernard edition. As it is to be expected, a number of manuscripts show minor variants, but except one which will be discussed later on, these variants are irrelevant for the argument. This gives us an ideal backdrop for our analysis.

First, a comparison of the mainstream text versus the Florence/Wellcome group. In the chapter on pityriasis, the editor rephrased the text, making it easier to understand, and slightly more vernacular ^15^. This is not an uncommon occurrence in medieval manuscript transmission – in fact, the two main principles a textual critic has to work with are *lectio brevior potior* and *lectio difficilior potior*, the shorter variant is the more likely (original) one and the more difficult variant is the likely (original) one ^16^. They also replaced a word that is slightly difficult to understand, namely ἀνενεχθείσης “brought up”, along with a preposition referring to it ^17^. This word forms part of a sentence explaining that pityriasis is caused by bad humours being “brought up around the head”. This sentence is not easily understood without any knowledge of ancient medical terminology, and it may at first sight give the impression that it was somehow corrupted in the course of the transmission. However, it was already extant in an important source of Theophanes, Paul of Aegina ^18^. The word used in Paul in the majority of manuscripts is ἀνενεχθείσης, with one witness reading ἀναχεθείσης “poured up”.

In the mainstream version, and in Paul, the phrase essentially means that bad humours are being excreted through the scalp, which then manifests in bran-like deposits forming on the surface of the skin. The editor of the Florence/Wellcome version rephrased this, stating that pityriasis is caused by “matter” going into the head. This makes the text easier to understand, but also fairly vague. The “bad humours” become neutral “matter”, and it is not clear that the matter is ultimately excreted through the skin. Even more problematic is that the editor then abruptly reverts to readings close to the mainstream text a few words later, saying that alternatively pityriasis may also be caused by two different humours, which obviously does not make sense ^19^. In the remainder of the passage discussed in this article, the editor just rephrases the text without any major changes to the content.

In the second paragraph, the editor of the Florence/Wellcome version made a number of changes to the text, which do not have any bearing on the content. For instance, they added a synonym, e.g. in the phrase διαβρωτικῶν τρωγόντων, two words meaning “eating away”. Otherwise, the text is slightly simplified, but not remarkably so. On two occasions, however, the editor makes decisive changes. At the beginning, they use the word αἰτία “cause”, to clarify that they are moving on from a description of the symptoms to an analysis of the causes of the disease. This is certainly correct, but not strictly necessary, as this would have been evident to a medically trained reader anyway. The second intervention is of a more drastic nature.

After a brief outline of the aetiology of this type of hair loss, i.e. an abundance of three possible humours, the mainstream version advises to assess the colour of the skin to determine which humour it is, and then administer the correct treatment. A colour resembling black or white would necessitate one specific treatment, a more pale colour another. Here, the editor of the Florence/Wellcome version adds another option after “more pale”, namely yellow. Here, they use a late, and somewhat vernacular, word to describe yellow, κίτρινον ^20^. This word is not extant in the corresponding passage in Paul of Aegina ^21^, which was the source for the mainstream version. It is, however, extant in one of the manuscripts transmitting the mainstream version, Ro1, which replaces the word “paler” with “yellow”. But, to complicate matters, Ro1 comes from an entirely different end of the stemma of the Theophanes tradition than the Florence/Wellcome version, and none of the manuscripts within its branch share the reading κίτρινον ^22^. Therefore, Ro1 cannot be the source for the Florence/Wellcome version, unless one assumes some form of contamination ^23^.

Consequently, this small detail turns out to be quite significant, as it appears in two ends of the transmission independently. Curiously, it is absent from both the source of Theophanes and some other texts that are connected to *De Curatione:* neither of the two versions of Ioannes archiatrus talk about yellow skin ^24^. This text goes back to a source that was very close to Theophanes. Moreover, the chapter on alopecia in Leo medicus (another text somewhat related to Theophanes) does not discuss the colour of the skin at all ^25^. Alexander of Tralles, whose *Therapeutics* share some links with the wider transmission of Theophanes, discusses skin colour in his chapter on alopecia, but does not mention any yellow ^26^. The source for all these authors, including Theophanes and Paul, is, directly or indirectly, Galen's vast and influential work *Method of Healing*, who equally does not mention yellow ^27^.

Next, I am going to examine the relationship between the mainstream transmission and the Escorial manuscript. The first part of the chapter on pityriasis is almost identical, except for one synonym. However, the editor suddenly adds some important information further on. Rather than just saying that “one” should remove the offending humour from the body, the editor specifies that this should be done by a “doctor”. The editor then adds a sentence to clarify the structure of the chapter, and the final sentence of the excerpt is rephrased, but without any major changes to the content covered.

In the second chapter, the editor of the Escorial manuscript rephrased the first part of the text, again without making any major changes to the meaning. As a result of these changes, the Escorial version is easier to understand than the mainstream text. The remainder of the text is almost identical to the mainstream.

The final manuscript to be discussed, Palermo XIII C 3, is of a different nature. It is not so much a rephrased text than a rearranged one. According to its title on f. 121r, the section of the manuscript contains an *iatrosophion*. The title is followed by a pinax, or table of contents, and then by the beginning of Theophanes' *De curatione*. On f. 145v to 146r, respectively, it contains the chapters on pityriasis and alopecia discussed in this article without any major alterations. This is then followed by some more chapters from Theophanes, roughly up to chapter 18 (Bernard) and f. 148v respectively. From f. 149r the text has been taken from another source. So in essence, an editor excerpted some chapters from Theophanes and used them to form part of a new handbook ^28^.

## Results and conclusions

Overall, what does the analysis of these three versions contribute to our research question? What was the motivation behind these changes? As for the first two versions discussed, a general trend was quite clearly to simplify the text, which could manifest in a slightly more vernacular wording. It is not difficult to guess the motives behind a mere simplification: the editor tried to make the text more accessible. Adding redundant synonyms had the same purpose. If a user did not understand one way of phrasing something, then perhaps he would have been able to understand the other. All this could be done while remaining well within the syntax and lexicon of the learned elite of the time. The motive to adjust the text a bit more to the vernacular does, on the other hand, raise a number of important questions. First of all, one is left wondering who the intended audience of these versions was. And then, it also raises the question of the date at which these changes were made.

As for the date, Theophanes lived, according to his own words ^29^, in the first half of the tenth century, which we can confidently use as a terminus *post quem* ^30^. The terminus *ante quem* is the fourteenth and sixteenth century respectively, as this is the date of the manuscripts. During this period, the Greek vernacular was already in existence; this can be determined from a few vernacular words transmitted in other sources, such as for instance Leo medicus on the medical side ^31^, but we are not in a position to reconstruct the development of the vernacular fully because of a lack of sources ^32^. Overall, though, it is safe to say that a slight adjustment towards the vernacular fits well into the general linguistic background of the time.

A slightly simplified and slightly more vernacular version would be more accessible to users who are literate, and educated enough to understand the classicising learned Byzantine idiom that was used in writing, but who were more comfortable with an idiom that was closer to the Greek spoken in everyday life. It is, however, well worth bearing in mind that the idiom was indeed just very slightly adjusted towards the vernacular. The editor did not translate the text.

Both editors had at the very least basic medical training along with some philological skills. Even though the process of redacting the text seems basic and straightforward to us, it does actually take some confidence and determination to prepare, in the eyes of the editor, an improved version of a text. As far as their medical training is concerned, both the editors of the Florence/Wellcome and the Escorial version add some new information – the former the word *aitia* “cause”, and the latter “doctor”. This shows at least a basic understanding of medicine and a familiarity with the structure of medical handbooks.

We cannot be certain where the word “yellow” originated, which was added in the Florence/Wellcome version. Perhaps it was already in existence in the manuscript that was used by the editor; alternatively, it may have come from a common, possibly even oral source such as an oral teaching tradition ^33^. In any case, the addition of an identifiable colour such as yellow – the word κίτρινον means “lemon coloured” – makes the text more precise. In Classical Greek, names of colours are notoriously difficult to interpret, and in many cases we can only guess what they may have referred to. In Galen's description of the scalp colour in alopecia patients, the words black and white are used – this much is clear – and then the rather unspecified “pale”. This terminology was very much the state of the art in Galen's time. We cannot be entirely certain about the Greek Middle Ages, as more basic research needs to be carried out on the development and characteristics of this idiom.

The Palermo manuscript presents a somewhat different picture, as far as the use and modification of sources is concerned. Here, someone compiled a text using more than one manuscript, and we cannot be certain about the reasons behind this decision. It is equally possible that the editor only had fragmentary sources at their disposal and then decided to stitch it all together to form a manual ^34^. Alternatively, they may have made a conscious decision to select certain passages from one source and others from another source. In any case, the way it was done reveals that we are yet again dealing with an editor who had at least basic medical training – they knew how to structure a therapeutic handbook – and who also had some philological skills.

So altogether, the analysis has shown that three different people with similar skill sets worked on and with Theophanes' text. They were confident handling the material, yet they were not part of the top range of brilliant scholars of the time. What we can see here is a more intermediary layer of abilities. Their attitude towards an author such as Theophanes was very different from the attitude of ordinary scribes who aimed to preserve the works of earlier authorities as accurately as possible.

## Data availability

All data is presented in the main text.

## Footnotes

1 First of all I would like to acknowledge all those who supported me in my research leading up to this publication. The manuscript work for this article was carried out during a Wellcome Trust University Award (091648/Z/10/Z). Dionysios Stathakopoulos and Petros Bouras-Vallianatos invited me to give a presentation at the Byzantine Studies conference in Belgrade, where this paper was presented. Moreover, I am grateful to the libraries who provided scans of manuscripts, and in particular if the material appeared open access on the internet. The same applies to any journal or archive that published primary and secondary sources in an open access format. In this revised version of the article, I am also able to thank the reviewers for their helpful comments. Some clarifications have been added to the main article, and other matters are addressed in a comment.

2 There are numerous examples for this practice throughout the field. Just one such occurrence would for instance be the preface of Ioannes archiatrus as discussed on p. 31 Zipser, B. 2009. *John the Physician's Therapeutics. A Medical Handbook in Vernacular Greek*. Leiden/Boston: Brill.

3 The date is indicated in the title of the work, where it is dedicated to emperor Constantine Porphyrogenitus. The best edition available is: I. Bernard 1794–95. *Theophanis Nonni Epitome de curatione morborum graece ac latine*. 2 vols., Gothae/Amstelodami: C.W. Ettinger. http://www.biusante.parisdescartes.fr/histoire/medica/resultats/?intro=nonnos&statut=charge&fille=o&cotemere=32590 An older edition: H. Martius 1568. Noni medici clarissimi, De Omnium particularium morborum curatione, ed. Martius. Argentorati: Josias Rihelius.

4 The main basis for the present article was Sonderkamp's comprehensive catalogue of the manuscript tradition of Theophanes, where you will also find detailed information on catalogues and relevant secondary literature: J. Sonderkamp, J. 1987. *Untersuchungen zur Überlieferung der Schriften des Theophanes Chrysobalantes (sog. Theophanes Nonnos)*. Bonn: Dr. Rudolf Habelt. Some, but very few updates have appeared on the Pinakes database published by the IRHT Paris. http://pinakes.irht.cnrs.fr

5 See the respective entry on the Pinakes database, and these catalogues for further information: Miller, E. 1848. *Catalogue des manuscrits grecs de la bibliothèque de l'Escurial*. Paris: A l'Imprimerie Nationale, 132. This book has been digitized. https://archive.org/stream/bub_gb_iiIDAAAAYAAJ#page/n1/mode/2up Revilla, Alejo, *Catálogo de los códices griegos de la Biblioteca de El Escorial*, vol. 1, Madrid : Imprenta Helénica, 1936, p. 506-507.

6 This manuscript has been digitized along with its catalogue entry, which appeared in: Bandini, A.M. 1770. *Catalogus Codicum Graecorum Bibliothecae Laurentianae*. Vol. 3, Florentiae: Typis Regiis, 147–151. A digitized version of this catalogue can be found on the Laurenziana website and in other locations. Some insights in the provenance of the manuscript can be found in Speranzi, D. 2010. 'Michele Trivoli e Giano Lascari. Appunti su copisti e manoscritti greci tra Corfù e Firenze', *Studi Slavistici* 7, 263–297. This article is available open access. http://www.fupress.net/index.php/ss/article/view/9212/8710

7 This manuscript has been digitized. http://wellcomelibrary.org/item/b19620214#?c=0&m=0&s=0&cv=0&z=-0.6713,-0.0736,2.3426,1.4715 A detailed catalogue entry can be found in: Bouras-Vallianatos, P. 2015. 'Greek Manuscripts at the Wellcome Library in London: a Descriptive Catalogue', *Medical History* 59, 2, 275–326. This article is available open access. http://journals.cambridge.org/abstract_S002572731500006X Sonderkamp was not aware of this manuscript.

8 For a detailed catalogue entry see the Manus Online website. http://manus.iccu.sbn.it//opac_SchedaScheda.php?ID=171784 This manuscript was not known to Sonderkamp, and it was first pointed out to me by Irene Calà, to whom I am very grateful. It has since also appeared on the Pinakes database of the IRHT. See also: Garzya, A. 2003. 'Pour l'édition des *iatrosophia* démotiques', in A. Garzya, J. Jouanna (eds.), *Trasmissione e ecdotica dei testi medici greci. Atti del IV Convegno Internazionale Parigi 17–19 maggio 2001*, Napoli: D'Auria, 165–171.

9 The Wellcome manuscript contains a substantial amount of mistakes, which are not extant in the Florence manuscript. While a number of these could be corrected by a scribe, this would certainly not be possible for all mistakes. The Wellcome manuscript also contains a *pinax*, or table of contents, which is not extant in the Florence manuscript. Notably though, this pinax reflects the text as it presents in the Florence and Wellcome version, which is, compared to the mainstream version, missing the preface. The Florence manuscript on the other hand is almost entirely free of mistakes, let alone serious mistakes, and it does not contain a *pinax*. For these reasons, I described the logical relationship between the manuscripts as “siblings” in the first version of the present article. In theory, it would be possible for the scribe who copied the Wellcome manuscript to copy its text from the Florence manuscript and to compile a *pinax* from scratch based on the main text body, however, given the low quality of the Wellcome manuscript, one is at least left wondering whether the scribe would have been able to do so. The possibility that the Florence manuscript once contained a *pinax*, which was then ripped out can be discounted. In conclusion, there is evidence to assume that the manuscripts are siblings rather than that one descended from the other. Because of the absence of substantial mistakes in the Florence manuscript, which would be the ultimate proof, I decided to change the main text of my article to “closely related and probably siblings” as suggested by a reviewer, and instead added this explanatory footnote.

10 This is easily explained; firstly, recipes are more likely to be corrupted in the process of copying. For instance, one can easily skip an entire recipe, or leave out a part of it by mistake, because these recipes are mostly lists of ingredients along with measures. So for instance one could skip from one mention of “two ounces” to the next leaving out text in between. Then, it was quite common for scribes or users of the book to add alternative recipes in the margins of the manuscript. These could then be included into the main text by a consecutive scribe. One can find examples for these phenomena in the transmission of any Byzantine therapeutic text.

11 Very few manuscripts transmit ἀνεχθείσης or similar, for instance the Palermo manuscript discussed in this article, which is clearly a misreading. Bernard prints this reading as well.

12 For the sake of clarity, Sonderkamp's sigla are used. Ro1 stands for Vat. gr. 292, 1-68v.

13 The specific challenges in translating Greek colour adjectives will be discussed later on in this article. In the following, ὠχρὸν will be translated as “pale” and κίτρινον as “yellow”, as these are very widely used standard translations of these words.

14 For instance, he calls pityriasis πιτυρίδα, which is the vernacular form of the word.

15 In the transcription of manuscript sources, the original spelling was kept as far as this was practicable.

16 There are a number of introductory works on the methods of textual criticism available. A very sharp and useful one would be West, M. 1973. *Textual criticism and editorial technique applicable to Greek and Latin texts*. Stuttgart: Walter de Gruyter.

17 This preposition, παρά, is somewhat prone to be corrupted in the course of transmission, as two other prepositions use a similar abbreviation.

18 Paul of Aegina III 3, 1 (= I 134, 18). Heiberg, I. 1924. *Paulus Aegineta*. Vol. 2, Lipsiae/Berolinae: In aedibus B.G. Teubneri (= CMG IX 2). http://cmg.bbaw.de/epubl/online/cmg_09_01.php

19 The problem is that according to the Florence/Wellcome version pityriasis is caused either by matter going into the head, or by some specific humours. This leaves the reader wondering what this “matter” may have been. The mainstream version talks about bad humours, which are then specified as two, or three, depending on how one reads the text, possible alternatives. Only the mainstream version makes sense.

20 A full text search on the Thesaurus Linguae Graecae database for κιτριν- yields only one text that can confidently be dated to Classical times, namely the third century AD, Cassius Dio 61, 10. Here, it is used to describe wood of a lemon tree. The word means “yellow” in modern Greek. Because only very few vernacular sources from Byzantine times have been edited, and because of the general difficulties in identifying colours, our analysis will have to come to an end here. The precise identification of the plant, from which this colour adjective derives is equally problematic. It could either be a lemon or a citron, both of which look extremely similar. The word λεμóνι *lemoni* from which the modern Greek word for lemon derives, is only very poorly attested. Various forms or derivates of κίτρον *kitron* are, on the other hand, much better attested. However, we cannot be sure whether the authors were consistent in naming these plants.

21 Paul of Aegina III 1, 1 (= I 129, 14 Heiberg).

22 A comprehensive analysis of the transmission of *De curatione* will be published in my forthcoming edition of the text. It was not included in this article as it would take up a significantly disproportionate amount of space.

23 This would be rather unlikely, given that there is no other evidence to suggest contamination, neither here nor in other chapters that were examined to establish the stemma of the mainstream version. On the contrary, the transmission of the mainstream version neatly falls into subgroups that ultimately form a clear bipartite stemma.

24 Ioannes archiatrus ω 90, 2 and 74, 1 respectively. Zipser, B. 2009. *John the Physician's Therapeutics. A Medical Handbook in Vernacular Greek*. Leiden/Boston: Brill.

25 Leo medicus *Synopsis Iatrikēs* VII 20 (= 213–215 Ermerins). Ermerins, F. 1840. *Anecdota Medica Graeca*. Lugduni Batavorum: Apud S. et J. Luchtmans, 81–221. https://archive.org/stream/anecdotamedicag00graegoog#page/n100/mode/2up

26 Alexander of Tralles *Therapeutics* I 1 (= I 441 Puschmann). Puschmann, Th. 1878. *Alexander von Tralles, Original-Text und Übersetzung*. Vol. 1, Wien: Wilhelm Braumüller. https://archive.org/details/b24755990_0001

27 Galen *Method of Therapy* XIV 18 (= X 1016 Kühn), as referenced by Puschmann in his notes on Alexander's *Therapeutics*. C. Kühn 1825. *Claudii Galeni Opera Omnia*. Vol. 10, Lipsiae: Prostat in officina libraria Car. Cnoblochii. http://www.biusante.parisdescartes.fr/histoire/medica/resultats/?cote=45674x10&do=chapitre Aetius Amidenus VI 55 (= II 199 Olivieri) also follows Galen in this respect. Oliveri, A. 1950. *Aetii Amideni libri medicinales*. Vol. II, Berolini: In aedibus Academiae Litterarum (= CMG VIII 2). http://cmg.bbaw.de/epubl/online/cmg_08_02.php

28 This practice is not uncommon in Byzantine medical literature, and this is by far not the only instance where excerpts from Theophanes have been used to build another collection, see for example Iviron 214. Yet, the Palermo manuscript presents the most refined and systematic approach to this end, which is why I decided to include it in my argument.

29 Theophanes dedicates his work to Constantine Porphyrogenitus, who reigned in the first half of the 10 ^th^ century AD. See p. 4 Bernard.

30 All versions described here clearly go back to the mainstream version directly, as they are dedicated to Constantine Porphyrogenitus. The only manuscript that does not preserve this piece of information is MSL 135; but since it is extant in its sibling, this is clearly secondary.

31 See for instance Leo medicus *Synopsis Iatrikēs* VII 23 (= 215 Ermerins). This text has been transmitted in a tenth century manuscript, the Par. Suppl. gr. 446. Leo sometimes adds vernacular synonyms to his text.

32 The most extensive evidence on the medical side is transmitted in the ω version of Ioannes archiatrus, amongst others in an early 14 ^th^ century manuscript, MSL 14, which gives us a neat and reliable time stamp. At this point, the vernacular was definitely in existence.

33 A few Byzantine medical texts that have come down to us give the impression that they may have been meant to be memorized. These are for instance a medical compendium in verse form attributed to Ideler, I. 1841. *Psellus: Physici et medici graeci minores.* Berolini: Typis et impensis G. Reimeri, 203–243. And a brief description on haematology on p. 293 of the same volume. Snippets of these rather formulaic texts could have been transmitted orally as well. For the latest state of research on Psellus see: Bouras-Vallianatos, P. 2015. 'A New Witness to Michael Psellos' Poem “On Medicine” (“De Medicina”)'. *Jahrbuch der Österreichischen Byzantinistik* 65, 9–12.

34 This would be supported by the fact that the manuscript contains *lacunae* on f. 153v, 154v, 157v, 160v, 189r and 209v.
