# Supplementary material for: Revisions of Theophanes Chrysobalantes De Curatione
Source: Wellcome Open Res. 2017 Apr 10;2:17. Originally published 2017 Mar 13. [Version 2] doi: 10.12688/wellcomeopenres.9991.2 (PMC5384629; doi:10.12688/wellcomeopenres.9991.2)
Supplement: Peer review history

# Peer review: Referee response for version 2

**DOI:** 10.21956/wellcomeopenres.12278.r21744

**Published:** 2017-04-18

**Authors:** Sophia Xenofontos

**Affiliations:** 1 Department of Classics, University of Glasgow, Glasgow, UK

I have read this submission. I believe that I have an appropriate level of expertise to confirm that it is of an acceptable scientific standard.

# Peer review: Referee response for version 2

**DOI:** 10.21956/wellcomeopenres.12278.r21743

**Published:** 2017-04-11

**Authors:** Maria Mavroudi

**Affiliations:** 1 Department of Classics, University of California/Berkeley, Berkeley, CA, USA

I have read this submission. I believe that I have an appropriate level of expertise to confirm that it is of an acceptable scientific standard.

# Response

**Published:** 2017-04-11

**Authors:** Barbara Zipser

**Affiliations:** Royal Holloway, University of London, UK

**To all four referees:**

First of all many thanks for your input. I would like to address a few points from the reviews in a comment rather than in the main text body of my article.

The question about the historical context of the transmission is a very good one, and MC already provided all there is to say in her review.

The reason why I kept my discussion of the Palermo manuscript brief, and focused on the Theophanes passages in question is because a PhD student in Germany is working on this manuscript. We divided the topic between us so that there is not going to be any undue overlap.

The “vernacular”:

I use the word “vernacular” to describe the medieval, and to a certain extent also early modern, idiom (that was mostly) spoken in the Greek world. This term is generally understood in anglophone scholarship, but it is far from ideal for all the reasons outlined by the reviewers. However, until a more fitting term is found, I shall continue to use “vernacular”.

We have only very few edited testimonies of texts that are solely written in the vernacular, but many more exist. Some more evidence can be extracted from other sources, such as for instance Leo medicus, as referenced in my article. The pronunciation of the vernacular is for instance described in Latin in some lemmata of Simon of Genoa's *Clavis Sanationis*.

There are indeed a number of major studies in the history of the Greek language. However, these studies all use different terminology to describe what I call the vernacular. Here are some samples:

- R. Browning 1969. *Medieval and Modern Greek*. Cambridge: CUP. On p. 55, he talks about informal, living speech and subliterary texts representing an uneasy balance between the purist ideal and the speech of the people.

- G. Horrocks 1997. *Greek. A History of the Language and its Speakers.* London: Longman. On p. 153-155 he talks about vernacular Greek.

- F. Rodriguez Adrados 2005. *A History of the Greek Language. From its Origins to the Present*. Leiden: Brill. On p. 245 he talks about popular texts.

A good analysis can be found in: D. Holton, I. Manolessou, “Medieval and Early Modern Greek”, in E. Bakker (2005). *A Companion to the Ancient Greek Language*, Chichester: Blackwell, p. 539-563. Many thanks to Petros Bouras-Vallianatos to pointing this out.

The general debate about the nature of modern Greek is also ongoing, which is certainly going to have an influence on the discussion on the medieval vernacular.

Altogether, it is quite clear that the question of diglossia in the Middle Ages cannot be resolved here, but perhaps the matters raised in the reviews might help further the discussion.

The “mainstream” version:

I use the word “mainstream” to describe the most frequently transmitted version of a medical text.

The odd παρά and the syntax of the versions:

I have thought about this a lot, when I wrote the article, and then again when I read the review, and I came to the conclusion that I shall leave it as it stands, and also in particular as we would need a better understanding of the medieval grammar and lexicon to resolve this.

The syntax has indeed been simplified in the versions.

The colour κίτρινον *kitrinon*:

It is notoriously difficult to identify colours in ancient or medieval texts. This is in particular the case as only very few sources have even been edited. Surprisingly, a TLG search did indeed yield one good source. It is transmitted in the Par. gr. 2329 f. 184v, a 17 ^th^ century manuscript (starts from line 3 of the second paragraph). http://gallica.bnf.fr/ark:/12148/btv1b10722308m/f189.item The language of the text could very well be consistent with an earlier, medieval date.

Καὶ κρόκον βάλε καὶ κορκουμὰν, καὶ μέλι, καὶ ἄλλα κίτρινα· νόει κρόκους ὠῶν καὶ χολὴν βοὸς κιτρίνου ξηράν. - And add saffron and turmeric and honey and other things that are *kitrina*. Consider egg yolk and the dry bile of a *kitrinou* bull.

This recipe is mentioned in connection with a procedure to make a metal appear like gold, and it is fairly clear that the colour the author had in mind was yellow.

Citron or lemon:

These modern English words designate two distinct types of fruit. However, it would be problematic to assume that these two words consistently described two distinct types of fruit in Classical and Medieval Greek as well. Citrons and lemons look very similar indeed, and it is certainly possible that these names were not used consistently. This would also be supported by the fact that the word for lemon is derived from the Greek word for citron in at least two different European language that immediately come to my mind, German and Polish.

We would need to gather more Greek textual evidence to address this question. What is currently available on the TLG does not suffice, and the situation may be complicated by regional dialects as well.

But perhaps it is worth pointing out that both words are relatively rarely attested on the TLG, which in turn raises a lot of questions.

Theological texts:

For some reason, theological scholarship does not have a problem with texts that were augmented later on, for instance *catenae. *For this reason, I adopted some editorial methods from the Nestle Aland edition of the New Testament for my edition of Ioannes archiatrus.

# Peer review: Referee response for version 1

**DOI:** 10.21956/wellcomeopenres.10767.r20944

**Published:** 2017-03-27

**Authors:** John Wilkins

**Affiliations:** 1 Classics and Ancient History, University of Exeter, Exeter, UK

This is a valuable and well researched article of a proper scientific standard. Title, abstract and structure are fine. Conclusions are well drawn.

 It concerns the history of medicine in the Byzantine period, a rich but seriously under-researched period, apart from the history of manuscripts, which is the topic of the article.

 The article shows that the scribes in certain parts of the tradition were modifying the text and simplifying it for their own purposes, in contrast with the accurate reproduction of the text in the scholarly branch of the tradition. This process is to be expected in Byzantium and throughout the other cultures - Syrian, Arabic, Hebrew and Western European - which adopted the Greek medical corpus.

 Minor suggestions I would have for the author are:

 Check the use of *kitrinon* in texts of the period. The citron is the citrus plant most likely to be familiar in Byzantium, more so than in texts in the time of Theophrastus or Galen. Its taste and flavours are not lemony any more than an apple tastes like a pear, nor is the colour lemon-yellow. I think it is more green-yellow and quite different from standard yellow words in Greek.

 In the first extract, the author might add, by way of explaining the simplification of the text, that a clause has replaced a genitive absolute. Is it a grammatical simplification as well as a muddled scientific modification?

 I very much like the conclusion that the scribes were medically literate and had a scientific interest in where or not a 'doctor' was needed, whether or not 'bad humour' was the medical term that was appropriate, and whether or not a Galenic 'cause' should be inserted into the text. These findings need to filter through to the medical and social history of Byzantium.

I have read this submission. I believe that I have an appropriate level of expertise to confirm that it is of an acceptable scientific standard.

# Peer review: Referee response for version 1

**DOI:** 10.21956/wellcomeopenres.10767.r20943

**Published:** 2017-03-21

**Authors:** Sophia Xenofontos

**Affiliations:** 1 Department of Classics, University of Glasgow, Glasgow, UK

This is an interesting article, which has a persuasive argument revolving around the transmission of sections from Theophanes Chrysobalantes’ *De curatione*. It is an important piece of work not only for the useful insights it offers into the different versions of the mainstream text but mostly for its observations on the working methods of different editors, their intellectual background and intended audiences. It will hopefully (re)emphasise the need for similar research which must be conducted in this significant area of study focusing on the circulation and reception of Byzantine medical works.

 The article is suitable for publication in its current form. Some suggestions are provided below, which the author may want to consider and/or address:

- Page 2, paragraph 2: I think it might be worth clarifying how the term “mainstream” is used throughout. Does this refer to the most widely circulated version of the text?
- Page 2, paragraph 2: is *De curatione* a therapeutic manual only, or important for its value as a diagnostic manual as well?
- Page 2, paragraph 3: What are the exact folio numbers preserving Theophanes’ text in the Palermo manuscript?
- Page 2, note 3: Constantine Porphyrogenitus’ name needs to be referred to consistently (compared to its occurrence in notes 27 and 28). Also, it is worth consulting and citing the useful article by Joseph A. M. Sonderkamp (1984) ^1^, who provides a lengthy discussion on the addressee of Theophanes’ work.  
- Page 2, note 3: Is Bernard’s edition the only available edition of the text? If not, it would be worthwhile to refer to earlier[?] editions.
- Page 2, “Transcription of the mainstream text”: Why “transcription”? It is stated above that the mainstream text is printed in line with Bernard’s edition. So why not just say “edition of the mainstream text”? Furthermore, it would be helpful to provide references to page number and lines in Bernard’s edition for all the passages commented upon in the article.
- Page 2, passage 4 On pityriasis: Since the name of diseases (here and below) are transliterated, they should be given in italics for the reader’s convenience.
- Page 2, passage 4 “It happens when bad humours are brought up around the head”: παρά + accusative denotes movement towards a person or a thing, so maybe “to”/“into” the head (see Demetrakos s.v. παρά, 12 and LSJ s.v. παρά C.I.1).
- Page 2, passage 4, “…cleaning of the superfluous humour”: Better: “evacuation”
- Page 2-3, passage 8: The translation for “Εἰ μὲν ... καθαίρομεν” is missing.
- Page 3, passage 8, “the hair of the head falls out”: maybe “the hair falls out from the head”
- Page 2, passage 8, “and first remove it”: perhaps “and the first thing you should do is to purge it”
- Page 2, passage 8, “the aloe- and bitter”: rather “by means of aloe and bitter...” in line with “διὰ τῆς ἱερᾶς ἀντιδότου” of the previous sentence.
- Page 3, Transcription of the Florence/Wellcome group, passage 4 “on the other white and bran like skin of the flesh”: “on the rest of the skin of the white and bran-like flesh”
- Page 3, Transcription of the Florence/Wellcome group, passage 4 “…or from salty phlegm…” : “…consisting either of salty phlegm …”
- Page 3, note 7: it is worth mentioning that the Wellcome manuscript is not listed in Sonderkamp’s sigla.
- Page 6: How do you define the term “vernacular”? Any relevant literature? See, for example, Robert Browning (1983) ^2^.
- Overall, do we find similar editorial “alterations” in other sections of Theophanes’ *De curatione*? Please consider adding some further examples.

I have read this submission. I believe that I have an appropriate level of expertise to confirm that it is of an acceptable scientific standard.

## References

[1] SonderkampJ: Theophanes Nonnus: Medicine in the Circle of Constantine Porphyrogenitus. *Dumbarton Oaks Papers*.1984;38: 10.2307/1291492 10.2307/1291492

[2] BrowingR: Medieval and modern Greek. *Cambridge University Press*.1983;

# Peer review: Referee response for version 1

**DOI:** 10.21956/wellcomeopenres.10767.r20945

**Published:** 2017-03-20

**Authors:** Marie Cronier

**Affiliations:** 1 Institut de recherche et d'histoire des textes (IRHT), French National Center for Scientific Research (CNRS), Paris, France

This article considers in a concrete way the thorny question of the revisions medical treatises of the Byzantine era may have undergone. It takes as an example a treatise composed in Byzantium in the 10th century by a certain Theophanes Chrysobalantes, the *Epitome de curatione morborum*.

 Its approach is relevant. It takes two concrete examples, namely two chapters devoted respectively to the diseases *pityriasis* and *alopecia*, and compares their text as given by the reference edition (published at the end of the 18th century) and by three different revised versions, which B. Zipser identified. She shows that in these three versions, the text has been rewritten - on the one hand to adapt it to the linguistic evolutions of Greek language, and on the other hand to become easier to understand. The analysis is well conducted and well argued. The results are convincing.

 However, I have some remarks, which do not detract from the value of the conclusions but aim to make the article more precise:

1. Concerning the question of the evolution of Greek language in the Byzantine period and the birth of the so-called "vernacular" Greek, there must exist a bibliography in the domain of linguistics. I do not know it personally (being not at all competent in linguistics), but I presume it would be useful, in order to ensure the credibility of this argument, to mention some basic publications in this field. I can not conceive that no one has ever studied the evolution of Greek language in medieval time.
2. Concerning the dating of the revised versions and the conclusions of the article, it seems to me that the three revisions discussed here should be clearly distinguished.
  Generally, moreover, it would be interesting to provide the readers with some context on the manuscripts that contain these versions. This would help to find some data to answer to the important question asked by B. Zipser: "[...] one is left wondering who the intended audience of these versions was."
  Even without depth research, one can get information by taking into account the basic bibliography of the manuscripts. For example :
  - The ms. Escorial T.III.1 was copied towards the middle of the 16th century by Iakobos Diassorinos (as indicated in Revilla's catalog, see detailed remarks below). Given the biography of the copyist, it is probable that this took place in Western Europe; there may be information about this in the Masters thesis of P. García Bueno, *Los manuscritos griegos de El Escorial copiados por Jacobo Diasorino: estudio paleográfico y codicológico*, Madrid, Univ. Complutense, 2011 (which I have not read) ^1^
  Now, Diassorinos is well known as a forger of texts; also, he was quite interested in medicine. There is therefore a good chance that the revised version transmitted by the Escorial manuscript would be from Diassorinos himself. We can at least ask the question, especially as we do not find this version in any previous manuscript.
  - The Palermo manuscript comes from Crete, where it was realized in the first half of the 16th century, probably around 1525 (as said in the manuscript description of ManusOnline, cited by B. Zipser). This manuscript is a huge medical compendium (more than 1000 folios!), gathering material from many different sources. No other manuscript resembles it. Could we make the hypothesis that it was its own copyists who did the editing work? We can at least ask the question, since these men already seem to have accomplished a sort of editorial work, by gathering scattered information.
  - As for the Florence manuscript, it was copied towards the beginning of the second half of the 14th century in Constantinople (one of its copyists was identified as Crateros, one of the collaborators of the great scholar Nicephorus Gregoras - see D. Bianconi, 2008 ^2^, mentioned in the bibliography of the Biblioteca Laurenziana website). The London manuscript MSL 135, which contains a version very similar to that of the Florence manuscript, was copied in the East, in the middle of the 16th century (as indicated in the catalogue of P. Bouras -Vallianatos, mentioned by B. Zipser). Except if it descends from the Florence manuscript (a fact which must be stated clearly), it must go back to a common model. This common model, which must have been prior to the second half of the 14th century, was therefore still in the Orient towards the middle of the 16th century. The London manuscript therefore confirms that the revision it preserves, together with the Florence manuscript, goes back to the middle of the 14th century at the latest, and that it circulated in Constantinople and its region, where it may have been made. One could make this hypothesis for the origin of this revised version even if, actually, nothing proves it.
  There are therefore three versions which clearly differ from each other, both in a geographical and chronological point of view (Western Europe in mid-16th century; Crete in the first half of the 16th century; Constantinople and its region in the middle of the 14th century). The article would benefit from distinguishing them.

 

 These remarks are only suggestions for further researches; they do not call into question the quality of the article.

 Remarks concerning details:

- The first mention of the Escorial ms.: one could add the Library’s name : Escorial, Real Bibioteca, Τ III 1.
- Note 5: There exists a more recent catalogue : Revilla, Alejo (O.S.A.), *Catálogo de los códices griegos de la Biblioteca de El Escorial*, vol. 1, Madrid : Imprenta Helénica, 1936, p. 506-507. ^3^
- The first mention of the Palermo ms.: add the end folios of the text (as indicated in the online catalogue mentioned at note 8): f.121r -238v (unless this indication is erroneous).
- “The manuscripts from Florence and the Wellcome Library present a near identical text, and are therefore siblings”: one could imagine that the Wellcome ms. may be a copy of the Florence ms. Is this impossible?
- Note 6: it could be useful to add the link to the digitized images: http://mss.bmlonline.it/s.aspx?Id=AVg6Otf3ADdoerJpI7oR&c=VIII#/book  (where one can also find a bibliography about the ms.).
- Note 11: since Sonderkamp’s book is not easily accessible, the reader would benefit from the indication of the exact shelfmarks of the manuscripts corresponding to Sonderkamp’s sigla (for example: which ms. is “Ro1”?).
- "Transcription of the Florence/Wellcome group”: chap. 4: read (or say that you reproduce the ms.’orthography) μελαγχολ ικοῦ ; α ἵματος ; κενώσ ῃς ; chap. 8: λευκ ότερον.
- “Transcription of T III 1”: chap. 4: read (or say that you reproduce the ms.’orthography) ἐπιφ ανείας; ἀπότ ηξις ; μελαγχολ ικοῦ ; chap. 8: Ε ἰ δὲ.
- Paragraph beginning with "After a brief outline [...]": one could imagine that the replacement of the classical ὠχρότερον by the more recent κίτρινον can have occurred independently in many different manuscripts. It could be just a sort of linguistic update. So B. Zipser is right in presuming no special relation between the Florence/Wellcome version and the Ro1 ms.
- Paragraph beginning with “The final manuscript [...]”: Are Theophanes’ *Therapeutics* the same book as *De curatione*?
- Note 29: the reader would appreciate a few more explanations (namely that Leo explicitly quotes a “vernacular” word?). One can not understand this without having a look at Leo’s text.
- Last phrase: “Their attitude towards an author such as Theophanes was very different from the attitude of ordinary scribes who aimed to preserve the works of earlier authorities as accurately as possible”: this is not entirely true for all texts. While sacred texts and, in general, texts from famous and most known authors were (mostly) transcribed with respect, it is obvious that scientific and technical texts, above all those attributed to little known authors (to tell nothing about anonymous texts), were much more subject to changes and rewordings. Alas, Theophanes is not the only medical author in this case.

I have read this submission. I believe that I have an appropriate level of expertise to confirm that it is of an acceptable scientific standard.

## References

[1] Garcia-BuenoC: Los manuscritos griegos de El Escorial copiados por Jacobo Diasorino: estudio paleográfico y codicológico. *Complutense Madrid University (Thesis)*.2011; Reference source

[2] BianconiD: La controversia palamitica. Figure, libri, testi e mani. *Segno e Testo*.2008;6:337-376

[3] RevillaA: Catálogo de los códices griegos de la Biblioteca de El Escorial, vol. 1. *Madrid : Imprenta Helénica*.1936;

# Peer review: Referee response for version 1

**DOI:** 10.21956/wellcomeopenres.10767.r20941

**Published:** 2017-03-14

**Authors:** Maria Mavroudi

**Affiliations:** 1 Department of Classics, University of California/Berkeley, Berkeley, CA, USA

This is a solid and useful article, analyzing how and why revisions were introduced to a widely diffused medical text authored in the tenth century. Although the article's conclusions are drawn on the basis of a specific text and its versions in concrete manuscripts (as is appropriate), they can be applied more broadly to the manuscript transmission of other medical (and more broadly technical) texts.

 The article is acceptable as is,  but would gain in interest and depth if the author could add some discussion on the following:

1. In the four manuscripts under examination, what other texts is Theophanes Chrysobalantos copied with? Do these other texts present characteristics similar to the revised versions of Theophanes Chrysobalantos? In other words, can these manuscripts as integral objects (and not as vehicles for single texts) be construed as professional manuals for practitioners with a particular profile, perhaps reflected in the register at which most of the texts they contain is pitched?
2. In the Escorial manuscript, could the specification that a doctor has to empty the superfluous humor etc, help us understand anything at all about a possible division of labor between doctors and medical assistants? Of course, this one mention of a doctor is not enough. The manuscript could, perhaps, be scrutinized for more clues (which it may or may not contain).
3. What can physical characteristics of the codices (e.g. one or many hands, types of paper, date and type of binding, possessor's notes, etc), tell us about the post-Byzantine reception of Byzantine medicine (especially given that three of the four are sixteenth-century manuscripts)? Do these sixteenth-century manuscripts appear to have been created within an Ottoman or early modern European context? What does this tell us about the role of Byzantine medicine in early Ottoman or early modern European practice?

Inquiry in the above three directions may allow a more detailed sociology of the redactors, scribes, and users of Theophanes' text. 

 An issue that does not affect the argument and overall scholarly contribution of the article, but one that the author may wish to address if she chooses to introduce revisions is the following: the article's current discussion of what a vernacular register is and when it is introduced into writing is somewhat old fashioned (predicated on aligning developments in Greek with developments in other European languages and the rise of the European "vernaculars"). All languages with long written traditions have multiple written registers (closer or more remote from the high brow canon). When vernacularisms become manifest in written literature largely depends on how long the surviving written record is. Vernacularisms in written Greek are evident since the translations collectively known as the Septuagint, the compilation of the Christian gospels (canonical and apocryphal), the publication of the acts of the church councils, or the composition of best sellers of monastic literature, such as the Spiritual Meadow by John Moschos—in other words, considerably earlier than the tenth century. Given that the earliest surviving Greek medical manuscripts are from the tenth century (e.g. Paul of Aegina now in Paris) and that, in their overwhelming majority, date from the twelfth century and later, our ability to discern vernacularisms in the medical texts earlier than these dates is limited. But this does not mean that a linguistic vernacularization of medical texts was not part of earlier medical practice and training.

I have read this submission. I believe that I have an appropriate level of expertise to confirm that it is of an acceptable scientific standard.
